# Anaesthetics and plants: from sensory systems to cognition-based adaptive behaviour

**DOI:** 10.1007/s00709-020-01594-x

**Published:** 2021-01-19

**Authors:** František Baluška, Ken Yokawa

**Affiliations:** 1 IZMB, University of Bonn, Kirschallee, 53115 Bonn, Germany; 2 Faculty of Engineering, Kitami Institute of Technology, Hokkaido, 090-8597 Japan

**Keywords:** Adaptive behaviour, Anaesthetics, Cell biology, Plant cognition, Sensory systems

## Abstract

Plants are not only sensitive to exogenous anaesthetics, but they also produce multitudes of endogenous substances, especially when stressed, that often have anaesthetic and anelgesic properties when applied to both humans and animals. Moreover, plants rely on neurotransmitters and their receptors for cell-cell communication and integration in a similar fashion to the use of neural systems in animals and humans. Plants also use their plant-specific sensory systems and neurotransmitter-based communication, including long-distance action potentials, to manage stress via cognition-like plant-specific behaviour and adaptation.

*Motto*

Claude Bernard: ‘… what is alive must sense and can be anaesthetised, the rest is dead.’

(Bernard 1878; Grémiaux et al. 2014; Kelz and Mashour 2019).

## Introduction

Ever since Aristotle placed plants outside the realm of sensitive organisms due to their immobility and apparent lack of sensation, plants have been considered somewhere in between living and non-living systems (Lindsay 1876; Ingensiep 2001; Mesaroș 2014; Linson and Calvo 2020). Immanuel Kant considered plants close to inorganic minerals due to their apparently senseless nature (Ingensiep 2001, p. 310). Later, Erasmus, Charles and Francis Darwin, together with Wilhelm Pfeffer, Gottlieb Haberlandt, Bohumil Nemec and Jagadis Bose, contradicted these views and believed plants to be actively living organisms based on their plant-specific sensory systems (Francé 1905; Stahlberg 2006). In 2005, the plant neurobiology initiative was established in Firenze (Italy) and was met with opposing arguments stemming from the view that plants lack agency, cognition and conscious behaviour (Alpi et al. 2007; Taiz et al. 2019; Draguhn et al. 2020). To address such opposition, first, it has never been claimed that plants have true neurons or neuronal synapses. However, plant cells have many features and properties that were originally attributed to neurons (Baluška 2010), including excitable plasma membranes with voltage-gated ion channels that can support plant-specific action potentials and synaptic-like endocytic vesicle recycling. In addition, in the root apex transition zone, cell-cell adhesion domains structurally and functionally resemble neuronal synaptic domains and have thus been introduced as plant-specific synaptic domains (Baluška et al. 2005). Second, since the field of neuroscience started with studies of human and animal brains, thus many terms which were originally used in a brain-specific manner turned out to have more general applications. This causes issues with terminology and comprehension (Maher 2020) when similar and/or analogous molecules, structures and processes are discovered later in other organisms, especially in plants.

Sensory systems, agency, cognition, behaviour and adaptation are fundamental attributes of all living organisms (Kováč 2006; Baluška and Mancuso 2009; Baluška et al. 2018; Calvo et al. 2020a, b; Trewavas and Baluška 2011; Witzany and Baluška 2012; Leopold 2014; Baluška and Levin 2016; van Duijn 2017, Lamme 2018; Reber and Baluška 2020). This follows from Darwinian evolution, in which all organisms, extinct and current, are part of the same evolutionary systems and utilise the same biological life principles. Of course, there are significant differences in these fundamental attributes between different types of organisms, but organism-specific versions are present in all organisms (Bernard 1878; Baluška and Mancuso 2009, 2020a,b; Baluška and Levin 2016; Linson and Calvo 2020; Reber and Baluška 2020). If we continue to maintain a Darwinian evolutionary position (Eisenstein et al. 2016), we must expect that all organisms rely on these faculties. In words of William James, ‘… if evolution is to work smoothly, consciousness in some shape must have been present at the very origins …’ (James 1890, p. 149).

## Plants and anaesthetics

Plants are emerging as very sensitive organisms with respect to anaesthetics. Importantly, anaesthetics can prevent the movement of animals, humans and plants via their quick prevention of action potentials (for plants, see Volkov et al. 2010, 2014; Grémiaux et al. 2014; Hedrich and Neher 2018; Yokawa et al. 2018, 2019; Pavlovič et al. 2020). Anaesthesia in humans induces a loss of awareness, which could also be hypothesised to occur for plants. Sensory events received at the plasma membrane (Matzke et al. 2019) are first translated into bioelectric signals (Levin 2019), which are later transformed into chemical signals acting in networks present not only in animals and humans (DeWeese and Zador 2006; Vosshall and Carandini 2009) but also in plants (Hedrich 2012; Reyer et al. 2020).

**Fig. 1:**
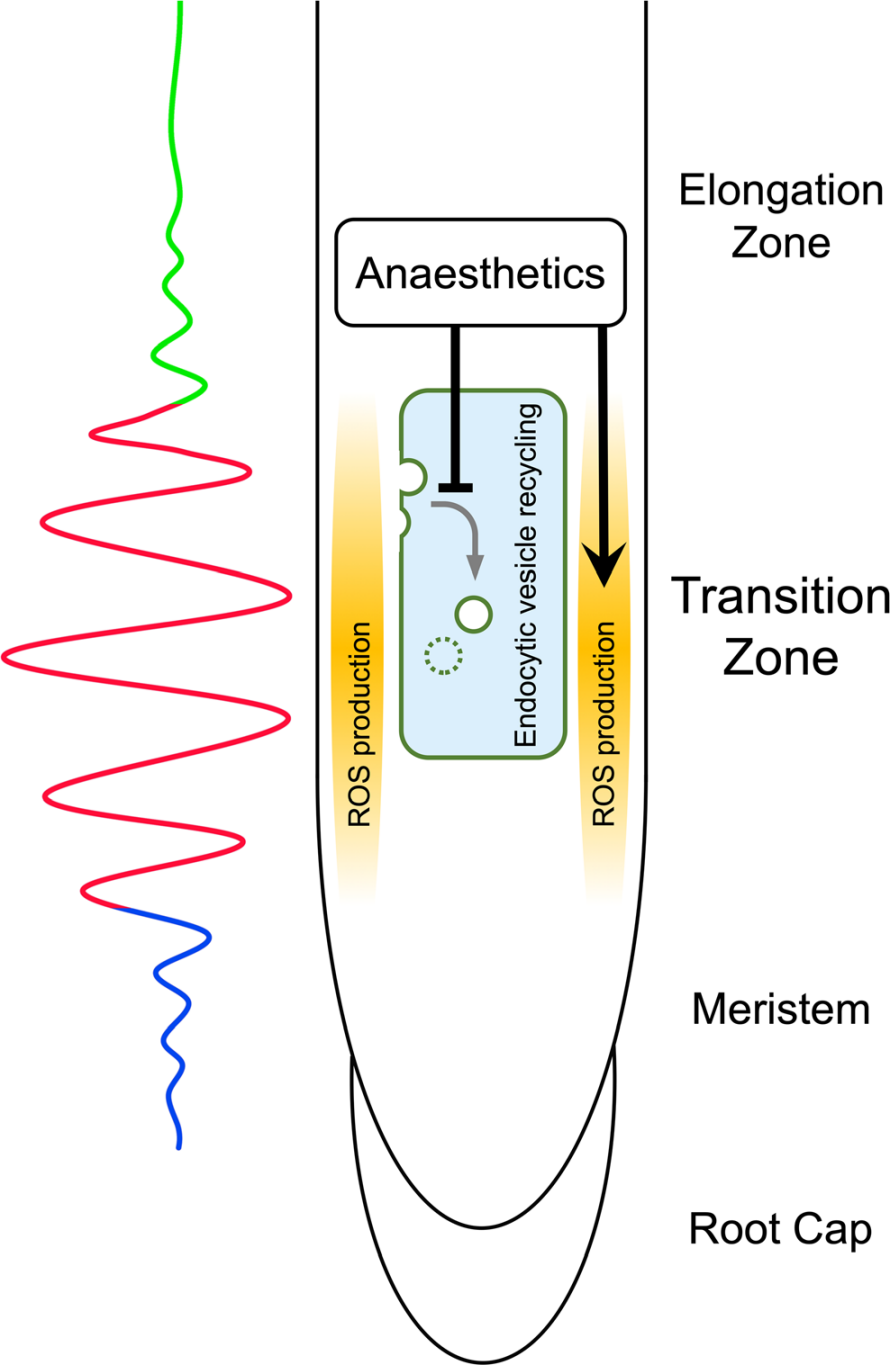
Schematic view of the root apex. The transition zone is the most sensitive root apex zone to anaesthetics which inhibit endocytic vesicle recycling and induce ROS imbalances (Yokawa et al. 2018, 2019). Cells in this bioelectrically active and oscillatory zone have high oxygen demand related to endocytic vesicle recycling (Baluška and Mancuso 2013) and are typical with high amounts of well-ordered lipid rafts (Zhao et al. 2015a,b). For more details, see Baluška and Mancuso (2013)

Draguhn et al. (2020) claim that anaesthetics affect numerous proteins and molecules. This seems to be true, but is not a relevant argument for the exclusion of plants in experimental studies aiming to illuminate the mysteries underlying their actions that lead to general immobility, weakening pain perception and a loss of consciousness. In addition, the induction of immobility by anaesthetics has the same biological basis in humans, animals and plants. As in animals, the blockage of action potentials stops organ movement in plants (Yokawa et al. 2018; Pavlovič et al. 2020). Plants have emerged as valid and relevant experimental systems in which to investigate the still-elusive primary anaesthetic targets that suppress plant action potentials (Yokawa et al. 2019). A recent paper reported that general anaesthetics target PLD2 localised to neuronal lipid rafts (Pavel et al. 2020). Anaesthetics are well known to associate with and to perturb lipid rafts (Weinrich and Worcester 2013; Kinoshita et al. 2019; Pavel et al. 2020). Importantly, PLDδ localises to lipid rafts in Arabidopsis (Xing et al. 2019), and there are several molecular tools and transgenic lines for use in studying the emerging roles of lipid rafts as cellular targets of anaesthetics (Li et al. 2012; Wang et al. 2015; Zhao et al. 2015a,b; Xue et al. 2018; Xing et al. 2019; Jaillais and Ott 2020). In root apices, cells in the transition zone (Fig. 1) show abundant lipid rafts with high lipid order under their cross-walls (Zhao et al. 2015a,b), which are active in the endocytic vesicle recycling (Baluška et al. 2005; Baluška and Mancuso 2013), as well as synchronous oscillations of several processes (Baluška and Mancuso 2013).

With respect to pain perception, it is obvious that plants do not perceive noxious stimuli in the same way as animals and humans. Obviously, plants do not have brain enclosed by a skull. However, in order to survive and adapt effectively, plants still need to be able to recognise what is dangerous and have evolved their own plant-specific sensory systems to do so. Anaesthetics are especially relevant in this respect, as plants produce a large variety of chemicals and substances when wounded or heavily stressed (Tsuchiya 2017) that have known anaesthetic effects on humans and animals (Baluška et al. 2016). For example, stress phytohormones such as ethylene and methyl salicylate are also anaesthetics (Campagna et al. 2003; Roohi and Imanpoor 2015; Baluška et al. 2016). Plant-derived menthol exhibits similar general anaesthetic effects as propofol (Watt et al. 2008). In addition, ethylene was used in human surgeries and emerged as a leading anaesthetic in the early twentieth century (Johnstone 1927; Dillard 1930), with its disuse in surgeries resulting from a series of accidents. Numerous other substances synthesised by plants, including diverse terpenoids, alkaloids and flavonoids, have anaesthetic activities (Tsuchiya 2017). Perhaps it is not surprising that ethylene and other plant-produced anaesthetics are particularly abundant in mature fruits (Baluška et al. 2016), which have been evolved by plants to be eaten by frugivores for the purpose of plant seed dispersal.

## Neurotransmitters and their receptors in plants

Ligand-gated ion channels underlie both excitatory and inhibitory transmission in animal nervous systems, in which glutamate and GABA act as specific ligands. Similarly, in plants, glutamate acts as an excitatory and GABA as an inhibitory ligand for plant-specific versions of glutamate and GABA receptors. More specifically, control of plasma membrane excitability is accomplished via glutamate and GABA in both animals and plants, where glutamate increases and GABA decreases membrane excitability via ion channel control (for plants see Ramesh et al. 2015, 2017; Žárský 2015). Glutamate and GABA receptors are often characterised as anaesthetic targets, and plant-derived bicuculline regulates mammalian GABA receptors as well as GABA signalling in plants (Ramesh et al. 2015, 2017; Žárský 2015). In addition, GABA acts as a ligand of the voltage-gated potassium channel GORK in plants (Adem et al. 2020), thereby shaping action potentials in plants (Cuin et al. 2018). Glutamate is also triggering action potentials in plants (Felle and Zimmermann 2007; Stolarz et al. 2010; Koselski et al. 2020). As with bicuculline control of GABA neurotransmission, glutamate neurotransmission can be controlled by plant-derived molecules such as berberine, an isoquinoline plant alkaloid (Lin et al. 2013). Interestingly, recent evolutionary analysis of glutamate receptors suggests that glutamate signalling involving plant glutamate receptors (Weiland et al. 2016; Wudick et al. 2018; Qiu et al. 2020) may have predated signalling via neuronal iGlur glutamate receptors in animals and humans (see Figure 1 in Stroebel and Paoletti 2020). Neuron-like electrical long-distance signalling in plants (Mousavi et al. 2013; Hedrich et al. 2016; Toyota et al. 2018; Muday and Brown-Harding 2018; Kumari et al. 2019; Reyer et al. 2020) assembles plant bodies into coherent units acting as single cognitive selves (Baluška and Mancuso 2020a). Beyond GABA and glutamate, plants also use a diverse array of other neurotransmitters for signalling and communication (Baluška et al. 2020).

## Sensory systems of plants support cognition-based and intelligent plant behaviour

Currently, it is accepted without a doubt that plants use exquisitely sensitive sensory systems to adapt to an ever-changing environment despite their immobility. Besides the unique plant senses that allow, for example, plant roots to navigate towards water or away from salty areas and light (Takahashi and Scott 1991; Galvan-Ampudia et al. 2013; Yokawa et al. 2014; Mo et al. 2015; Dietrich 2018), all senses known in humans and animals are present as plant-specific versions in plants (Francé 1905; Weiler 2003; Gagliano et al. 2017; Mescher and De Moraes 2015; Rodrigo-Moreno et al. 2017; Baluška and Mancuso 2016; Sopory 2019). Plant sensory systems thus support putative plant-specific versions of cognition and intelligence.

## Conclusions

Plants emerge as truly living organisms with all related cognitive and behavioural consequences. The use of anaesthetics promises to be an excellent tool for probing not only the possibility of cognition, and other (awareness) functions in plants, but also the elusive molecular targets of substances producing analgesic and anaesthetic effects in humans (Baluška et al. 2016; Kelz and Mashour 2019). Considering plants as organisms devoid of sensory and cognitive faculties has serious consequences for our technology-based exploitation of trees and forests, which are critical systems contributing to climate stability (Baluška and Mancuso 2020b). Preserving healthy forests is our prime task for preventing environmental collapses in the future. Understanding that plants are truly living organisms, with all the associated cognitive abilities, will help us to forge a transformation of the biological sciences, as well as to support the preservation of our current life-friendly climate.
